# Immune Modulatory Properties of Collagen in Cancer

**DOI:** 10.3389/fimmu.2021.791453

**Published:** 2021-12-08

**Authors:** Anne Mette Askehøj Rømer, Marie-Louise Thorseth, Daniel Hargbøl Madsen

**Affiliations:** ^1^ National Center for Cancer Immune Therapy, Department of Oncology, Copenhagen University Hospital - Herlev and Gentofte, Herlev, Denmark; ^2^ Department of Science and Environment, Roskilde University, Roskilde, Denmark; ^3^ Department of Immunology and Microbiology, University of Copenhagen, Copenhagen, Denmark

**Keywords:** cancer immunology, collagen, extracellular matrix, tumor microenvironment, immunotherapy, T cells, macrophages, matrix immunology

## Abstract

During tumor growth the extracellular matrix (ECM) undergoes dramatic remodeling. The normal ECM is degraded and substituted with a tumor-specific ECM, which is often of higher collagen density and increased stiffness. The structure and collagen density of the tumor-specific ECM has been associated with poor prognosis in several types of cancer. However, the reason for this association is still largely unknown. Collagen can promote cancer cell growth and migration, but recent studies have shown that collagens can also affect the function and phenotype of various types of tumor-infiltrating immune cells such as tumor-associated macrophages (TAMs) and T cells. This suggests that tumor-associated collagen could have important immune modulatory functions within the tumor microenvironment, affecting cancer progression as well as the efficacy of cancer immunotherapy. The effects of tumor-associated collagen on immune cells could help explain why a high collagen density in tumors is often correlated with a poor prognosis. Knowledge about immune modulatory functions of collagen could potentially identify targets for improving current cancer therapies or for development of new treatments. In this review, the current knowledge about the ability of collagen to influence T cell activity will be summarized. This includes direct interactions with T cells as well as induction of immune suppressive activity in other immune cells such as macrophages. Additionally, the potential effects of collagen on the efficacy of cancer immunotherapy will be discussed.

## Introduction

With a constantly growing knowledge about the extracellular matrix (ECM), it has become clear that the ECM is by no means an inert scaffold, but rather a dynamic structure that can regulate the function of cells in contact with it (1). This ability of the ECM to influence cellular responses has been a major focus area in the matrix biology research field within the last decades. In connection to cancer, the ECM has been studied extensively in order to elucidate exactly how it influences tumor progression and metastasis. The majority of these studies have focused on the ability of the ECM to modulate the behavior of cancer cells or to stimulate the malignant transformation of epithelial cells. In the field of cancer immunology, much less attention has been given to the ECM and its potential role in modulating immune cell activity. However, recent reports have shown that the ECM can indeed influence the behavior of immune cells, thereby launching a potentially new research field: matrix immunology.

## Extracellular Matrix Remodeling in Cancer

### Collagen Organization

The ECM is a complex network of various macromolecules surrounding the cells within the body. It is composed of two classes of macromolecules, the fibrous proteins and the proteoglycans consisting of core proteins with one or more glycosaminoglycans (GAGs) covalently attached (2, 3). The main types of fibrous proteins are elastin, fibronectin, laminins and collagens (3, 4). The ECM can be classified into two categories based on function; the basement membrane and the interstitial matrix. The basement membrane forms an anchoring sheet-like layer between the parenchyma and the connective tissue. It is mainly composed of collagen type IV, laminins, nidogen 1 and 2, and various proteoglycans such as perlecan (5). The interstitial matrix is the matrix surrounding the cells. It is composed of proteoglycans and various fibrous ECM proteins secreted mainly by fibroblasts within the stroma (3, 6). In cancer, fibroblasts are the main producers of the tumor-specific ECM, but other cells can also contribute. These cells include endothelial cells (7), cancer cells (8, 9), and immune cells such as macrophages (3, 5, 10). Besides secreting ECM components, fibroblasts can also exert tension on the matrix, organize collagens into sheets and fibers, and influence the alignment of the collagen (3).

Collagens are the main constituents of the ECM comprising around 30% of the whole protein mass in the body (3, 11). 28 types of collagens have been identified (12), which based on their structure and function can be divided into subtypes including fibril-forming, fibril-associated collagens with interrupted triple helices (FACITs), network-forming, transmembrane, endostatin-producing, anchoring fibrils and beaded-filament-forming collagen (13, 14). The fibrillar collagen type I is the most abundant type of collagen and the primary constituent of the interstitial matrix (3, 15).

Collagen is composed of three left-handed polypeptide chains called *α*-chains held together by inter-chain hydrogen bonds. These intertwine to form a right-handed triple helical structure. The *α*-chains are composed of an amino- and a carboxy terminus flanking Gly-X-Y repeats. Of these, X is often a proline and Y a hydroxyproline. Each type of collagen contains a unique combination of *α*-chains (16, 17). The main type of collagen, collagen type I, consists of two *α*1-chains and one *α*2-chain (16, 18). Collagens are initially transcribed and translated into pre-pro-polypeptides. These undergo multiple post-translational modifications in the endoplasmic reticulum (ER) and in the Golgi apparatus. Following this, the resulting procollagen is packed into secretory vessels and transported to the extracellular space (18, 19). In the extracellular space the procollagen is modified by proteases to remove the N- and C-propeptide domains, generating mature collagen units that polymerize to form insoluble collagen fibers (18). Lastly, cross-linking can be introduced by lysyl-oxidase (LOX) in order to generate larger collagen fibers (18, 20, 21), and increase the stiffness of the ECM (22).

### Collagen Degradation

The tight triple helical structure of collagen renders it very resistant to proteolytic cleavage. In fact, only a few proteases have been shown to be able to cleave native collagen type I under physiological conditions. These include members of the matrix metalloproteinase (MMP) family and the cysteine protease family. The MMPs capable of cleaving collagen are referred to as collagenases and include MMP-1, -2, -8, -13 and -14 (23). In collagen type I, the cleavage happens primarily at Gly^775^-Ile^776^ and Gly^775^-Leu^776^ in the α-1 and α-2 chain, respectively. This cleavage generates well-defined fragments ¼ and ¾ of the length of the full molecule (24). The exact molecular mechanism of MMP-mediated collagen cleavage has been excellently reviewed in (25). As opposed to the intact collagen, these fragments are unstable under physiological conditions and prone to degradation by additional proteases including MMP-2 and -9, the so-called gelatinases, as well as the serine protease fibroblast activation protein (FAP) (26–28). The proteolytically generated collagen fragments can also be internalized by receptor-mediated uptake and routed to the lysosomes for complete degradation by cysteine proteases (29–31). This process has been shown to be mediated primarily by two endocytic collagen receptors, the mannose receptor (MR)/CD206 and the urokinase plasminogen activator receptor-associated protein (uPARAP)/Endo180/CD280, which are expressed mainly by macrophages and fibroblasts, respectively (32, 33).

### Collagen Remodeling During Cancer Development

During tumorigenesis the ECM is extensively remodeled. The existing ECM is degraded and substituted with a tumor-specific ECM, which is often more linearized, of increased stiffness, and has a high collagen content. Degradation of the ECM involves the concerted action of multiple proteolytic systems and several different cell types of the tumor microenvironment (34). The degradation of collagen type I alone involves cleavage of collagen fibers by the collagenolytic MMPs MMP-1, MMP-8, MMP13, and MMP14 (MT1-MMP) which can be expressed by cancer cells or stromal cells (34). Complete collagen turnover involves subsequent lysosomal degradation upon cellular uptake of collagen fragments mediated by the MR or uPARAP (33, 35). In tumors, collagen internalization is primarily mediated by TAMs and CAFs, with M2-like TAMs being the dominant collagen-internalizing cell type (36, 37). CAFs are the central cell type responsible for production of collagen in the tumor microenvironment, although studies have shown that macrophages and cancer cells can also contribute to the production of collagen (36, 38). CAFs have recently been recognized as a group of several CAF subsets and especially one of these CAF subsets seems to be the main producer of collagen in the tumor microenvironment (39, 40). The newly synthesized ECM is characterized by being of high stiffness and density, and very rich in collagen. A high collagen-density and degree of collagen fiber alignment have been linked to a poor prognosis of several cancers. This includes breast cancer, pancreatic cancer, gastric cancer, and oral squamous cell carcinomas (41–45). The reason for this correlation is still not clear. The effects of collagen density and tumor stiffness on cancer cells have been investigated for more than a decade, and recently, studies have also investigated the effect of collagen on immune cells such as tumor-infiltrating lymphocytes (TILs) and tumor-associated macrophages (TAMs).

## Effects of Collagen Density on Cancer Cells

Increased stiffness compared to healthy tissue is a characteristic of most solid tumors that render them detectable by palpation. The high stiffness of tumor tissue has been shown to correlate with increased deposition of collagen as well as increased crosslinking of collagen fibers (46–49).

The high ECM stiffness is not only a passive bystander of cancer, but can also affect and drive many stages of tumor progression; from malignant transformation and increased metabolic adaptability to enhanced intravasation, facilitating metastasis (50). In healthy tissue, matrix stiffness also controls many important cellular functions, such as development and homeostasis (51). *In vitro*, substrate stiffness has been shown to affect naïve mesenchymal stem cells, which when cultured on soft matrices, mimicking brain tissue, commit to a neuron-like lineage while when cultured on rigid matrices, mimicking collagenous bone, commit to an osteogenic lineage (52).

In connection to cancer, the link between matrix stiffness and malignant transformation of epithelial cells has been investigated. Using mammary epithelial cells (MECs), Paszek et al. showed in a seminal study that increased collagen density led to perturbed morphology and disrupted basal polarity *in vitro* (53). They demonstrated that increased matrix stiffness drives a mechanoregulatory feedback loop in which focal adhesions (FA) are promoted through integrin aggregation. This in turn activates signaling pathways, increases cytoskeletal tension and further FA formation, promoting malignant transformation (53). This signaling loop was also demonstrated in a study by Provenzano et al. (54). In addition, they found that increased collagen density *in vitro* promoted an invasive phenotype of MECs and caused altered gene expression including upregulation of genes associated with proliferation (54). Increased matrix stiffness through collagen crosslinking was in another study showed to promote FA and induce invasion through enhanced signaling of PI3K (48). PI3K signaling has also been shown to be important for epithelial-mesenchymal transition (EMT) (55). When normal murine mammary gland epithelial cells were treated with transforming growth factor β (TGF-β), culture on soft matrices induced apoptosis while culture on stiff matrices led to EMT through the PI3K/Akt pathway (55). Increased matrix stiffness has also been suggested to modulate the metabolism of cancer cells. Specifically, culture of the metastatic breast cancer cell line 4T1 on a high-density collagen matrix resulted in an increased capacity to use glutamine as fuel source for mitochondrial respiration. This was not observed to the same degree for the non-metastatic breast cancer cell line 4T07 (56).

Metastatic disease requires the ability of a malignant cell to escape the primary tumor site by binding to vasculature and intravasate to reach a secondary location. Matrix stiffness has been shown to be of importance for this process. In response to increased stiffness, endothelial cells were shown to upregulate the protein cellular communication network factor 1 (CCN1), which activated β-catenin causing upregulation of N-cadherin on the surface of the endothelium. This was in turn shown to facilitate cancer cell-endothelium binding (57, 58). A high matrix stiffness can also induce epithelial-to-mesenchymal transition (EMT) in cancer cells, leading to increased metastasis (59). This process is dependent on stiffness-induced nuclear translocation of the transcription factor TWIST1 (59). LOX-induced collagen cross-linking is an important mediator of increased matrix stiffness in tumors and a driver of metastatic tumor growth (60, 61). Consequently, inhibition of LOX has been shown to reduce metastasis (61, 62).

Indirectly, a stiff matrix is also capable of supporting tumor progression by favoring growth of endothelial cells and thereby stimulating angiogenesis (63). Additionally, matrix stiffening promotes the activity of the transcriptional co-activators Yes-associated protein (YAP) and transcriptional coactivator with PDZ-binding motif (TAZ), which in turn are required for CAF-induced matrix stiffening, creating a positive feedback loop further driving the cancer-promoting effects described above (64).

## Collagen Can Affect the Immune Environment in Tumors

In addition to the effects on cancer cells, collagen has also been shown to affect tumor infiltrating immune cells. Of these, TILs and TAMs are of special interest due to their cytotoxic and anti-inflammatory activities, respectively.

### Collagen-Mediated Modulation of T Cell Activity

T cells are lymphoid cells that can be divided into several subsets depending on their T cell receptor (TCR) and expression of co-receptors, in particular CD4 and CD8. In the tumor microenvironment, especially the CD8^+^ T cells are of interest due to their cytotoxic activity. CD4^+^ T cells mainly acts to orchestrate the activity of other immune cells, but they can also have direct cytotoxic activity (65).

However, the ability of T cells to kill cancer cells is often suppressed in the tumor due to the existence of a highly immune suppressive tumor microenvironment. This tumor microenvironment is characterized by containing cells with an immunosuppressive phenotype including myeloid-derived suppressor cells (MDSCs), M2-polarized macrophages, and regulatory T cells (Tregs), and by the upregulation of several immune inhibitory molecules such as programmed death ligand 1 (PD-L1) and -2 (PD-L2), TGF-β, indoleamine 2,3-dioxygenase (IDO), and arginase 1 (ARG1) (66–69). Several studies have investigated the T cell inhibitory effects of these cellular components of the tumor microenvironment, but recently the effect of the ECM on T cell activity has also gained attention. ECM components, such as collagen, have been reported to directly or indirectly influence the migration, phenotype, and function of T cells.

#### Collagen Can Control the Migration of T Cells

Collagen in the tumor microenvironment can affect the ability of T cells to kill cancer cells by regulating the migration of T cells into the tumor. The organization of collagen in tumors is highly heterogenous but generally found to be more closely packed in the tumor periphery and more loosely packed within the center of the tumor. In addition, the collagen fibers are often aligned perpendicularly to the tumor boundary (42, 45). The density of collagen and the degree of collagen alignment are strong negative prognostic factors.

T cells can efficiently migrate in different environments including collagen matrices using an amoeboid migration mode (70). In 3D culture assays, T cells do however migrate slower through collagen gels of high density compared to low density (71, 72). This reduced migration speed was suggested to be a consequence of decreased pore size of the matrix (72). The increased stiffness associated with a higher collagen density could, however, also contribute to the reduced migration speed since matrix stiffness has also been shown to affect T cell migration (73, 74). In a study using optically tunable hydrogels, it was elegantly demonstrated that increased matrix stiffness in this assay system led to reduced T cell migration independently of the pore size (75). In contrast to cancer cells, which depend on protease activity for migration in a high-density collagen matrix, T cell migration in collagen is independent of proteolytic remodeling of the collagen fibers (72, 76). *In vitro* studies have also demonstrated that T cells preferentially migrate along the collagen fibers, indicating that the collagen orientation could control the migration of T cells (77).

The decreased T cell migration speed in a high-density collagen matrix as well as the ability of collagen fibers to guide T cell migration suggest that collagen in the tumor microenvironment could limit T cell infiltration. In agreement with these *in vitro* studies, migration along the collagen fibers has also been elegantly demonstrated using *ex vivo* culture of tissue slices from lung tumors and ovarian tumors (78–80). These studies confirm that the alignment of collagen fibers can limit T cell migration into the tumor core. Similar indications of collagen-mediated restriction of T cell infiltration were observed in a murine prostate cancer model as well as in human pancreatic cancer samples (77, 81).

#### Collagen Density Can Regulate T Cell Activity

**Figure 1:**
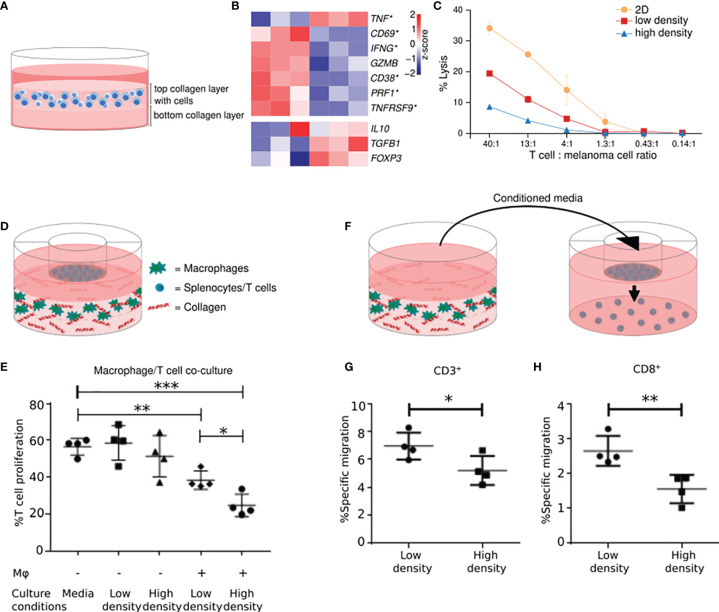
A high collagen density inhibits T cell activity and stimulates the immunosuppressive activity of macrophages. High-density collagen affects T cell activity. **(A)** T cells were transiently stimulated with PMA and ionomycin and subsequently embedded and cultured in a collagen matrix of high (4 mg/ml) or low density (1 mg/ml). **(B)** T cells were cultured in high-density- or low-density collagen matrices for 2 days and their gene expression profiles analyzed. Heatmaps of normalized (Z-score) RNAseq read counts of genes encoding markers of T cell activity (top panel) and Tregs (bottom panel). Significantly regulated genes indicated with asterisks. 6 out of 7 markers of T cell activity were significantly downregulated by high-density collagen culture. A trend towards an upregulation of Treg markers was observed. **(C)** Tumor infiltrating T cells were cultured for 3 days on plastic (2D) or in 3D high- or low-density collagen matrices. T cells were extracted and incubated for 4 hours with autologous melanoma cells in different ratios of T cells: melanoma cells. Lysis of melanoma cells by T cells was analyzed using a ^51^Cr-release assay, with addition of 10% Triton X-100 used for determining maximum lysis (100%). The cytotoxic activity of T cells was impaired by 3D culture, in particular high-density collagen culture. **(D)** RAW 264.7 macrophages were embedded and cultured in a collagen matrix of high- or low density. Splenocytes isolated from BALB/c mice were seeded in transwell inserts on top. After 3 days, T cell proliferation was analyzed using a BrdU-based flow cytometry assay. **(E)** Proliferation of T cells was decreased when co-cultured with macrophages compared to T cells cultured alone. The proliferation was further decreased when co-cultured with macrophages grown in high-density collagen compared to low-density collagen. **(F)** T cells were seeded in transwell inserts above conditioned medium from macrophages cultured for 3 days in high- or low-density collagen matrices. T cells were allowed to migrate towards the conditioned medium for 26-28 hours, and migrated cells were analyzed with flow cytometry. **(G, H)** T cells migrated significantly less towards conditioned medium from macrophages from high-density collagen matrices. The reduced migration was observed for CD3^+^ T cells **(G)** and for CD8^+^ T cells **(H)**. *P < 0.05; **P < 0.01; ***P < 0.001. **(A–C)** were adapted from (85). **(D–H)** were adapted from (86) with permission from *The American Association of Immunologists, Inc*.

T cell activation involves the formation of an immunological synapse between a T cell and an antigen-presenting cell (82). Older studies have shown that a collagen-dense environment can affect this interaction and reduce T cell activation (83, 84). Recently, we have identified that collagen density can also profoundly affect the activity of T cells after the initial activation phase (85). Specifically, cultivation of pre-activated T cells in a 3D high-density collagen matrix, mimicking tumor ECM, led to decreased proliferation compared to T cells cultured in a low-density collagen matrix. In addition, the T cells cultured in high collagen density upregulated Treg markers and downregulated cytotoxic T cell activity markers (**Figures 1A, B**). In alignment with these changes in the cells’ gene expression profile, we found that TILs cultured in 3D matrices of high collagen density compared to low collagen density were subsequently less capable of killing autologous melanoma cells, showing that collagen can directly affect the function of TILs (**Figure 1C**). Whole-transcriptome analyses indicated that the underlying mechanism of collagen-mediated modulation of T cell activity might involve autocrine TGF-β signaling (85). We did not investigate if the increased stiffness associated with a higher collagen density could be involved in the effects on T cell activity. The study of mechanosensing in T cells is still in its infancy, but an interesting study by O’Connor et al. showed that T cell activation was mitigated when cells were cultured on substrates of increasing stiffness (87). The effect included reduced proliferation and expression of cytokines associated with T cell activity. However, other studies of the effects of substrate stiffness on T cell biology, have not demonstrated a similar modulation of T cell activity (88, 89). The exact reason for these discrepancies is still unclear but could be a consequence of differences in substrates, T cell origin, and T cell stimulation between the different studies.

Collagen has also been shown to affect T cells *in vivo.* During tissue regeneration, implantation of collagen scaffolds into wounded muscles of mice promotes the formation of an immunosuppressive microenvironment (90). Specifically, collagen led to an increase in the CD4:CD8 ratio among the infiltrating T cells and the CD4^+^ T cells were skewed toward a Th2 phenotype. Additionally, the T cells in the collagen-implanted wounds expressed higher levels of anti-inflammatory cytokines (90).

#### Collagen-Receptors Expressed by T Cells

A possible way collagen can exert its effect on T cells is through the interaction with receptors on the surface of the cells. Several collagen binding receptors can be expressed by T cells. These include leukocyte-associated Ig-like receptor-1 (LAIR-1), discoidin domain receptor 1 (DDR1), and several integrins.

LAIR-1 is an immune-inhibitory transmembrane receptor with an Ig-like extracellular domain (91). It is a member of the Ig superfamily, and has been found to be expressed on the majority of PBMCs and thymocytes (92, 93). LAIR-1 is expressed by CD4^+^ and CD8^+^ T cells, with expression being highest in the naïve T cells (91, 94). Collagens are high affinity ligands for LAIR-1 (95) and both transmembrane collagens and secreted collagens, such as collagen type I, can bind to LAIR-1 on the surface of immune cells (96). For natural killer (NK) cells this interaction was shown to inhibit their cytotoxic activity (96). Using T cells isolated from LAIR-1 knockout mice, it has been demonstrated that collagen can inhibit TCR signaling in a LAIR-1 dependent manner (97). Additionally, studies have shown that cross-linking of LAIR-1 using anti-LAIR-1 antibodies directly inhibits T cell activity (91, 94). Further studies are needed to elucidate the role of LAIR-1 in the immunosuppressive tumor microenvironment, but based on current knowledge, LAIR-1 could be a promising cancer therapeutic target.

Another collagen binding receptor expressed on T cells is DDR1. The DDRs are a subfamily of receptor tyrosine kinases, consisting of DDR1 and DDR2, known to interact with a range of collagens (98). DDR1 consists of six isoforms and is primarily expressed by epithelial and carcinoma cells (99, 100). DDR2 is mainly expressed by cells of mesenchymal origin like fibroblasts and smooth muscle cells (101). A few studies have, however, shown that DDR1 expression is induced by TCR activation of T cells and plays a role in the migration of T cells through collagen matrices (101, 102). It is therefore speculated that DDR1 could be an interesting therapeutic target for improving T cell migration to the tumor (101).

Lastly, several integrins found on T cells are known to bind collagen. These include *α*_1_ *β*_1_ integrin and *α*_2_ *β*_1_ integrin (5, 103). These integrins are expressed on activated T cells and upon TCR stimulation they promote adhesion to the ECM (104). *β*_1_ integrins can also have co-stimulatory effects on antigen-stimulated T cells, rendering them more proliferative when cultured on surfaces coated with collagen type I compared to other ECM components such as fibronectin (105). This co-stimulatory effect was shown to be particularly potent in the case of collagen type I mediated activation of *α*_2_ *β*_1_ integrin (106). Also, *α*_1_ *β*_1_ and *α*_2_ *β*_1_ integrins expressed by activated T cells have been found to be important for the generation of an inflammatory response in a mouse model of delayed type hypersensitivity (107). Th17 cells express *α*_2_ *β*_1_ integrin, and the binding of collagen to this integrin leads to increased interleukin (IL)-17 synthesis (108). In the same study, it was shown that blockade of *α*_2_ *β*_1_ integrin decreases the severity of collagen-induced arthritis in mice (108).

### Collagen-Mediated Modulation of Macrophage Activity

Macrophages are myeloid cells belonging to the innate immune system. They are known to be very plastic cells, and dependent on their environment they can acquire an M1- or M2-polarized phenotype. The M1-polarized macrophages have a pro-inflammatory phenotype characterized by the expression of pro-inflammatory cytokines such as IL-1β, inducible nitric oxide synthase (iNOS) and tumor necrosis factor α (TNFα) and by the ability to present antigens on major histocompatibility complex (MHC) molecules. M2-polarized macrophages are anti-inflammatory cells expressing markers such as IL-10, TGF-β, and ARG1. However, newer studies have pointed out that this classification of macrophages is too simplified. Instead of macrophages being either M1- or M2-polarized, they are mostly somewhere in between, often expressing both types of markers simultaneously (109, 110).

TAMs are mainly M2-like anti-inflammatory macrophages with the ability to reduce a potent anti-tumor immune response. Consequently the number of TAMs is associated with a poor prognosis for several types of cancer (44, 111–114). TAMs can originate from tissue resident macrophages or from circulating monocytes that infiltrate the tumor (115). How TAMs acquire this pro-tumorigenic phenotype is still largely unknown, but several studies have shown that their migration and immune-suppressive activity can be greatly affected by the composition and mechanical properties of the surrounding ECM, and in particular by collagen (86, 116–118).

#### Collagen-Mediated Regulation of the Immune Suppressive Activity of Macrophages

In the tumor microenvironment, TAMs are often detected in close contact with collagen (119), and it is therefore an appealing hypothesis that the interaction with collagen could modulate the activity of the cells. This could happen during the differentiation of monocytes to macrophages or during the polarization towards an M2-like phenotype.

Cultivation of primary monocytes on a collagen type I coated surfaces has been shown to stimulate the differentiation to macrophages (120, 121), and during PMA-induced differentiation of the monocytic cell line U937, collagen type I causes reduced production of pro-inflammatory cytokines (122).

We have recently shown that 3D-cultured macrophages are directly affected by high collagen densities mimicking the ones found in tumors (86). Macrophages cultured in collagen matrices of high density compared to low density acquired a distinct expression profile, which included the differential expression of immune-regulatory genes and genes encoding chemokines. Co-culture assays revealed that macrophages cultured in high-density collagen inhibited the proliferation of T cells more than macrophages cultured in low-density collagen (**Figures 1D, E**). Additionally, the gene-expression changes were associated with a decreased ability to attract CD8+ T cells (**Figures 1F–H**). Altogether these findings illustrate that the surrounding collagen density can instruct macrophages to become more anti-inflammatory (86).

In alignment with these findings, culture of monocytes on decellularized matrices from colorectal tumor tissue or from normal colorectal tissue strikingly showed that tumor matrices drive monocytes towards M2-polarization (123). This could be a consequence of the higher collagen density of tumor matrices compared to the matrices from normal tissue (123). Similarly, decellularized matrices from obese and lean breast tissue were used to examine the effects of these matrices on macrophage function (124). Obesity was associated with increased amounts of interstitial collagen, and when bone-marrow derived macrophages (BMDMs) were cultured on the matrices derived from obese tissue, they acquired both morphological and genetic characteristics similar to those of M2-like TAMs (124).

In addition to these studies of primary monocytes/macrophages, the human monocytic cell line THP-1 has also been shown to acquire an M2-like phenotype when cultured in a gelatin-based hydrogel compared to regular tissue-culture conditions (125). In another study using PMA-stimulated THP-1 cells cultured in collagen gels of low or high density, it was, however, unclear if an increase in the surrounding collagen density stimulated M2-polarization of these THP-1 derived macrophages (126).

The exact reason why macrophages cultured in high-density collagen seem to acquire an M2-like phenotype still needs to be elucidated. One of the underlying mechanisms could involve the increased stiffness associated with an increased collagen density.

Studies have shown that cultivation of primary macrophages on stiff ECM surfaces can affect their migration speed, morphology, proliferation, and phagocytic activity (127, 128) and make them acquire a more M2-like phenotype (128, 129). Additionally, it has been shown that cultivation of THP-1 derived macrophages on stiff surfaces increases the expression of M2-markers compared to culture on softer surfaces (130). However, other studies have shown that macrophages can also upregulate the expression of M1-markers when cultured on stiff surfaces (131–133). The opposing results could be due to the different range of stiffness, type of coating, cell types, and stimulation used. For example, one study found that cultivation of unstimulated BMDMs on hydrogels of increased stiffness led to upregulation of M2-markers, whereas the same cell type stimulated with lipopolysaccharide (LPS) downregulated M2-markers and upregulated M1-markers on stiffer surfaces (133). Another example of how macrophages of different origin and activation state respond differently to ECM changes was shown by Stahl and colleagues (134). They found that pulmonary macrophages isolated from patients with idiopathic pulmonary fibrosis acquired a more M2-like phenotype when cultured on various types of collagen compared to pulmonary macrophages isolated from healthy donors (134). The mechanosensing ability of macrophages has been suggested to involve the ion channel transient receptor potential cation channel subfamily V member 4 (TRPV4) (128, 132) and the transcriptional coactivator YAP (135).

Several studies have indicated that collagen can stimulate M2-polarization of macrophages *in vivo*. For instance, implantation of collagen gels into injured muscles of mice resulted in an increased amount of M2-like macrophages compared to saline injected control mice (90). This effect was, however, shown to be dependent on collagen-induced Th2-polarization of CD4^+^ T cells (90). During skin wound healing in mice, it was also demonstrated that collagen injected into wounds led to M2-polarization of macrophages. This effect appeared to be mediated by the acid-sensing pathway-associated lysosomal adaptor protein, Lamtor1, indicating that phagocytosis of collagen and subsequent lysosomal signaling could be critical for the observed M2-polarization (136). In rats, implantation of crosslinked collagen disks likewise resulted in increased accumulation of M2-like CD206^+^ macrophages (137).

#### Collagen-Receptors Expressed by Macrophages

Several collagen-binding receptors are expressed by macrophages and could be involved in the cellular response to the surrounding collagen.

DDR1 can be expressed by macrophages and affect their cellular functions. DDR1 mRNA has been detected in human monocytic cells and the expression of DDR1 increases upon activation with IL-1β, granulocyte-macrophage colony-stimulating factor (GM-CSF), LPS, or phytohemagglutinin (PHA) (138). The role of DDR1 for macrophage function has been addressed in studies using DDR1-overexpressing THP-1 cells (138, 139). Here it was reported that the DDR1 isoforms DDR1a and DDR1b increased the adherence to collagen coated plates in a β1-integrin independent manner (138). Additionally, DDR1a promoted the ability of THP-1 cells to migrate through 3D collagen matrices (138). Cultivation of DDR1b-overexpressing THP-1 monocytes on collagen-coated surfaces increased the expression of inflammatory markers such as IL-1β, IL-8, MIP-1a, and MCP-1 compared to mock-transfected control cells (139). In the murine macrophage cell line J774A.1, collagen induced iNOS expression and consequently nitric oxide production in a DDR1-dependent manner (140). Murine Kupffer cells have also been shown to express DDR1, and pre-treatment of these cells with collagen led to an increased ability to attract cancer cells in a DDR1-dependent manner (141).

Several types of integrins are expressed on macrophages, with the β2-integrin family being the most common (142). However, macrophages can also express the collagen-binding α2β1-integrin (142). This has been demonstrated for primary macrophages isolated from the peritoneum of mice, and for these cells α2β1-integrin is essential for adhesion to collagen (143). In a recent study, it was furthermore shown that α2β1-integrin mediated the migration and mechanosensing of macrophages cultured on 3D collagen matrices upon deformation by contracting fibroblasts (144). α2β1-integrin is also expressed by the monocytic cell line, THP-1, where it is involved in M2-polarization induced by 3D culture in a gelatin-based hydrogel (125).

LAIR-1 is expressed by the majority of myeloid cells, including monocytes and macrophages, and it has been reported to have inhibitory effects on their cellular functions (93). In monocytes, LAIR-1 ligation with an agonistic antibody inhibited TLR-mediated activation (145). Additionally, cultivation of M1-stimulated murine or human macrophages on surfaces coated with LAIR-1 ligand peptide has been shown to result in decreased secretion of the pro-inflammatory cytokine TNFα as well as T cell attracting chemokines (146). Recently, it was demonstrated that LAIR-1 also promotes the differentiation from classical to non-classical monocytes in the bone-marrow (147). Surprisingly, this study also showed that global or myeloid-specific deletion of LAIR-1 in mice increased experimental lung metastasis of B16-F10 melanoma (147). Further studies are needed to elucidate the role of LAIR-1 in other cancer models and its role in modulating anti-tumor immunity.

Osteoclast-associated receptor (OSCAR) is another collagen-binding receptor, which is expressed in osteoclasts in mice, and in monocytes, macrophages, dendritic cells, and osteoclasts in humans (148–150). It is a member of the leukocyte receptor complex that associates with FcRγ, and through FcRγ-signaling OSCAR is critical for osteoclastogenesis (150, 151). In contrast to LAIR-1, OSCAR-signaling has mainly been associated with immune activation (35). In dendritic cells derived from human blood monocytes, OSCAR was shown to trigger cellular activation events (152), and in monocytes it potentiates the pro-inflammatory response of Toll-like receptor (TLR) ligands (153). This effect was confirmed in another study, where collagen stimulated the release of pro-inflammatory cytokines from monocytes in an OSCAR-dependent manner (154).

Macrophages also express the MR, which is an endocytic receptor with multiple ligands including collagen (155, 156). The receptor binds collagen and proteolytically generated collagen-fragments and promotes cellular internalization of the collagen for lysosomal degradation (32, 157, 158). *In vivo*, this process of MR-mediated collagen internalization has been demonstrated by macrophages in the skin (37, 159) and in tumors (36, 160), and genetic deletion of MR leads to an accumulation of collagen in murine tumors (36). It is not yet known if the MR-collagen interaction can lead to changes in macrophage activity and function.

## The Role of Collagen for Immune Activity in Tumors *In Vivo*

Several studies have attempted to elucidate the role of collagen for cancer progression and metastasis *in vivo*. However, only relatively few of these studies specifically investigated the effects of collagen on immune cell infiltration and activity.

The transgenic Col1a1^tm1jae^ (Col^R^) mice have a mutation in the collagenase cleavage site of the α1 chain of collagen type I, and as a consequence of the reduced collagen turnover they accumulate collagen in various tissues. These mice were interbred with MMTV-PymT mice, which spontaneously develop mammary cancer, and used by Keely and colleagues to investigate the effect of increased collagen density on mammary cancer progression and metastasis. The large collagen accumulations observed in the tumors of these mice were accompanied by increased tumor growth and metastasis (161). In another study by the same group, it was demonstrated that the increased breast tumor growth in the collagen accumulating transgenic mice was associated with increased infiltration of macrophages, neutrophils and α-smooth muscle actin (*α*SMA)-positive fibroblasts (162). The collagen-dense tumors were additionally characterized by an increased expression of cyclooxygenase-2 (COX-2) and prostaglandin E2 (PGE2). Blocking of COX-2 with Celecoxib decreased collagen deposition and infiltration of macrophages and neutrophils, suggesting that COX-2 modulates tumor progression in collagen dense tumors (162). In a follow-up study, the cell composition of the tumor microenvironment was examined by flow cytometry (163). No significant changes in cell composition were observed although a trend towards increased neutrophil recruitment was seen. Additionally, the cytokine profiling indicated a change in neutrophil activation. A potential role of neutrophils was confirmed by blocking the recruitment of these cells, which limited the increased tumor growth in the collagen-accumulating mice (163). Another study using orthotopically injected breast cancer cells, showed no difference in primary tumor growth between Col1a1^tm1jae^ and wildtype (wt) mice, but significantly more metastasis to liver and lung in the collagen accumulating mice (164). The tumor-promoting effects of collagen observed in Col1a1^tm1jae^ mice was also supported by a study from Northey et al. that showed increased proliferation of MECs indicative of a higher risk of breast cancer development (165). These seminal studies were proceeded by another well-performed study, which used Col1a1^tm1jae^ mice combined with a chemically induced or transplanted model of hepatocellular cancer (166). In this study the Col1a1^tm1jae^ mice were, however, found to develop significantly fewer and smaller tumors compared to wt mice (166). The observed tumor growth reduction in Col1a1^tm1jae^ mice was suggested to be a consequence of the lack of proteolytically generated collagen fragments that stimulate integrin-signaling (166).

Recently, three studies have investigated the role of collagen for tumor progression, using conditional collagen type I knockout (KO) mice. Chen et al. inactivated the *Col1a1* gene in αSMA^+^ fibroblasts and combined this with a genetically induced model of pancreatic ductal carcinoma (PDAC) (167). In this model system, they observed a 50% reduction in stromal collagen type I and, interestingly, an accelerated tumor growth and decreased overall survival compared to control mice. The tumors in conditional Col1a1 KO mice had increased infiltration of CD206^+^;F4/80^+^;Arg1^+^ myeloid cells and lower levels of B- and T cells (167). These changes in the immune microenvironment were suggested to be part of the reason for the observed effects on tumor growth. Two other recent studies used a similar approach to study the effects on *Col1a1* inactivation in CAFs or in the entire liver of mice with intrahepatic cholangiocarcinoma or liver metastases (168, 169). The conditional collagen type I KO mice did not show altered primary tumor growth, but did show increased growth of liver metastases (168, 169). In the liver metastases, no differences in immune infiltration and inflammatory markers were found, apart from a decrease in *Cd4* and *Foxp3* mRNA levels (169). Instead the authors proposed that the anti-tumorigenic effect was due to collagens ability to physically restrict tumor expansion (169).

The results are in line with a previous study where SPARC^-/-^ mice, which display reduced deposition of fibrillar collagen, were used to study the effects of collagen on pancreatic cancer growth and liver metastasis. Tumors in SPARC^-/-^ mice had decreased levels of collagen type I and III and higher levels of TAM-infiltration, which were associated with reduced survival and increased metastasis (170). Another study used mice with impaired pro-collagen processing to show that collagens produced selectively by the cancer cells had anti-tumorigenic effects (171).

In these *in vivo* studies of the effects of collagen on tumorigenesis and metastasis, it was not investigated how collagen deposition in the tumor affects the efficacy of cancer immunotherapy. In one study it was, however, shown that depletion of αSMA^+^ fibroblasts reduced the amount of collagen, and that this was accompanied by increased efficacy of anti-CTLA-4 therapy (172).

Lastly, studies have directly or indirectly investigated the effects of LOX-mediated collagen cross-linking on tumor growth in mice. Treating early stage PDAC with a LOX-inhibitor together with the chemotherapeutic drug gemcitabine, increased the overall survival of the mice by reducing metastasis. Interestingly, the combination therapy led to an increased number of macrophages and neutrophils in the primary tumors (173). However, another study reported that LOX knock-down using shRNA reduced the number of CD11b^+^ cells in breast cancer lung metastases (174).

In late stage murine PDAC, a LOX-inhibitor did not have any effect on survival of the mice but, interestingly, tumors were found to have an increased number of T cells and decreased number of neutrophils (173). It could thus be of interest to further combine this treatment with immunotherapy.

**Figure 2:**
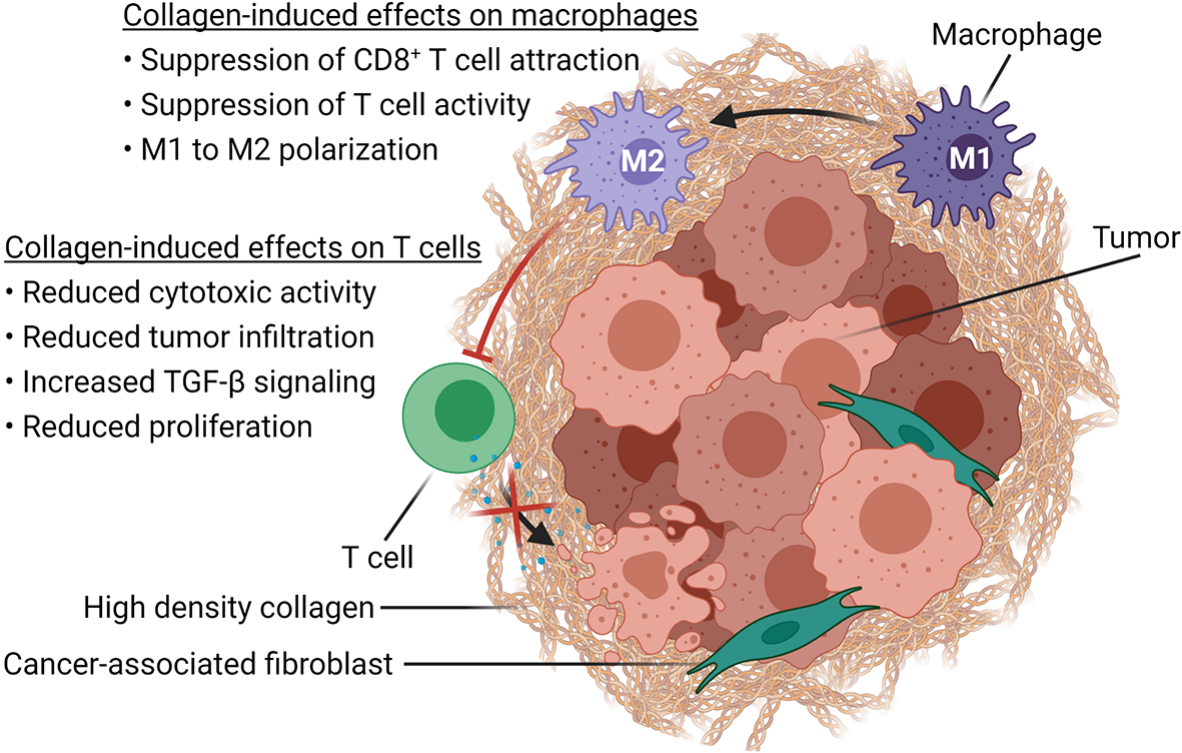
Immune modulatory effects of high-density collagen in cancer. Schematic representation of how increased collagen density, associated with tumor progression, affects immune cells in the TME. High-density collagen drives M1 to M2 polarization of macrophages, which in turn suppresses CD8^+^ T cell attraction as well as T cell activity. High-density collagen also affects T cells directly by increasing TGF-β signaling and by reducing tumor infiltration, proliferation and cytotoxic activity. Created with BioRender.com.

Based on *in vitro* and *in vivo* studies, collagen is likely influencing anti-tumor immune responses by directly modulating T cell activity as well as through the regulation of macrophage activity (**Figure 2**).

## The Effects of Tumor Collagen on Immunotherapy

Immunotherapy is a promising new type of cancer therapies using the ability of the body´s own T cells to kill cancer cells. However, a large number of patients do not respond to these treatments. As outlined in this review, collagen could be a novel target for improving the efficacy of immunotherapies such as checkpoint inhibitors (74, 175–180), TIL-based therapy (181), and cancer vaccines (182). The hypothesis that collagen in the tumor microenvironment can affect the outcome of cancer immunotherapy is supported by studies that have identified the serum level of the N-terminal pro-peptide of collagen type III (PRO-C3) as a marker of poor prognosis following anti-CTLA-4 and anti-PD-1 checkpoint inhibitor therapy in metastatic melanoma patients (183, 184). This serum marker is believed to reflect the presence of a highly collagen-dense tumor microenvironment (185).

In lung cancer, a high collagen level has also been shown to correlate with reduced efficacy of anti-PD-1/PD-L1 therapies (175). In mouse models of lung cancer, anti-PD-L1 resistance was shown to be associated with enhanced deposition of collagen, as well as fewer and more exhausted tumor-infiltrating CD8^+^ T cells. The effect of collagen on CD8^+^ T cells was mediated by LAIR-1, and combining anti-PD-1 with blockade of LAIR-1 significantly increased the therapeutic efficacy (175). Blockade of LAIR-1 has also been shown to increase the number of tumor-infiltrating CD4^+^ and CD8^+^ T cells and enhance the efficacy of anti-PD-L1 treatment in humanized murine xenograft models of several cancers, including colon- and pancreatic cancer (176, 177). In another study, it was shown that reduction of tumor stiffness in mice using a LOX-inhibitor increased the number of tumor-infiltrating T cells and improved the response to anti-PD-1 therapy (74). Reduction of collagen deposition in tumors, has also been obtained through inhibition of focal adhesion kinase (FAK) in murine models of pancreatic cancer (181). In addition to reducing collagen-density in tumors, inhibition of FAK decreased the number of infiltrating anti-inflammatory immune cells such as TAMs and Tregs, and increased the number of CD8^+^ T cells (181). These changes were associated with significantly improved efficacy of both TIL-based therapy and checkpoint inhibitor therapy (181). Recently, the use of a bacterial-based agent for delivery of collagenase to murine pancreatic tumors was demonstrated (186). This approach led to reduced collagen levels in the tumors and, importantly, also to enhanced efficacy of checkpoint inhibitor treatment (186). The ability of collagen to influence immunotherapy could involve DDR2-signaling since treatment of several murine cancer models with a combination of anti-PD-1 antibody and a DDR2-inhibitor led to an increase in CD8^+^ T cells and a reduced tumor burden (187). Inhibition of TGF-β has also been shown to reduce collagen levels in tumors (188–190) and to improve checkpoint inhibitor therapy (180, 190). The exact mechanism of action of this blockade is, however, difficult to delineate because of the multiple roles of TGF-β including direct effects on immune-suppressive cells in the tumor microenvironment (191).

The negative influence of collagen on cancer immunotherapy, was not observed in a study by Elisseff and colleagues where they co-injected B16-F10 melanoma cells and urinary bladder matrix (UBM) scaffolds into mice (192). These scaffolds, which are characterized by high levels of collagens, decreased tumor growth and improved the response to anti-PD-L1 or anti-PD-1 treatment (192). The tumor microenvironment of these UBM-associated tumors was characterized by increased number of CD4^+^ T cells and NK cells, and fewer Tregs compared to control tumors (192). It should, however, be noted that these UBM-scaffolds do not only consist of collagen but also contain other ECM-components, which could have multiple biological roles.

Collagen can interact with a number of ECM proteins, and consequently the level of collagen in tumors could correlate with the levels of other ECM proteins as well. This situation has been observed for hyaluronan, for which an increasing intratumoral content was accompanied by an increasing collagen level (193). Consequently, it cannot be excluded that immune modulation associated with high collagen levels could also be a consequence of altered levels of other ECM components.

## Conclusion

The ability of the ECM to influence immune cell behavior constitutes a novel research field, which could be termed matrix immunology. Here we have reviewed the current knowledge about the ability of collagen to directly or indirectly affect T cell activity. The majority of the reviewed studies focus on collagen type I, which is the most abundant of the collagens. It should, however, be noted that other less abundant collagen types could have different effects on the activity of immune cells. In addition to collagen, the ECM also contains many other components that all potentially could influence the cells in contact with it. Some of these components, such as versican, extracellular matrix protein-1 (ECM1) and hyaluronan, have already been suggested to have direct immune modulatory function (194–196). The current knowledge about the ability of different ECM components including hyaluronan to modulate immune activity has been excellently reviewed by (197). However, for most ECM components it is still unknown if they can influence immune cell activity. The immune modulatory functions of the ECM could influence the development and progression of cancer as well as the outcome of cancer therapies. Consequently, future studies within this field could reveal targets for new cancer therapies. Finally, it should be noted that the importance of the ECM in regulating immune activity extends beyond the cancer research field, since the dysregulation of immune activity is a key feature of multiple other pathological conditions.

## Author Contributions

AR, M-LT, and DM wrote, edited, and approved the final version of the manuscript. All authors contributed to the article and approved the submitted version.

## Funding

This work was supported by the Lundbeck Foundation (grant number R307-2018-3326, DM), the Danish Cancer Society (grant number R174-A11581-17-S52, DM), the Dagmar Marshalls Foundation (DM and AR), the Jens og Maren Thestrups legat til Kræftforskning (AR), the Agnes and Poul Friis’ Fond (AR), the Herlev Hospitals Forskningsfond (AR), and the Else og Mogens Wedell Wedellborgs Fond (AR).

## Conflict of Interest

The authors declare that the research was conducted in the absence of any commercial or financial relationships that could be construed as a potential conflict of interest.

## Publisher’s Note

All claims expressed in this article are solely those of the authors and do not necessarily represent those of their affiliated organizations, or those of the publisher, the editors and the reviewers. Any product that may be evaluated in this article, or claim that may be made by its manufacturer, is not guaranteed or endorsed by the publisher.
